# Indents

**Published:** 1902-06-15

**Authors:** 


### INDENTS.

**☞ Brief Articles of Technical or Scientific Value will receive notice and comment. Contributions invited.**

FORMALDEHYDE ANTIDOTE.—Give fifteen to twenty drops of aromatic spirits of ammonia well diluted in water. Other ammonia preparation may be used.—Merck.

A USEFUL HEMOSTATIC.—Cotton soaked with oil of turpentine and pressed into the tooth socket after extraction will check the hemorrhage promptly.—*Medical Summary*.

PHENOL AND COCAIN AS AN OBTUNDENT.—Dr. N. S. Jenkins recommends the use of a twenty percent solution of cocain, with carbolic acid as the solvent, applied hot to make excavating sensitive dentin tolerable.—*Cosmos*.

TO COLOR ARSENICAL PASTE.—Add five percent of lamp black to arsenical paste, and it will be easy to detect any portion which gets on the tooth or outside of the cavity in applying. It does not help or hinder the action of the arsenic.—G. V. Black, in *Review*.

FOR PERIOSTITIS.—The following is recommended for periostitis: Iodin crystals are dissolved in absolute alcohol until it is completely saturated. A mixture is then made of one-third of this solution, one-third of aconite tincture, and one-third of chloroform.—*British Journal of Dental Science*.

Chloroform Deaths Following Attack of Influenza.—Wm. Caldwell draws attention to the importance of obtaining a history of recent attacks of influenza before administering chloroform. Many deaths result from this source of nervous weakness and consequent depressed heart function. —*Dental World.*

Chloretone as a General Anesthetic.—Chloretone is used in physiological laboratories as a general anesthetic for animals, instead of ether and chloroform. A single dose of 0.2 gramme per kilo of body weight is sufficient to profoundly anesthetize a dog for hours. The drug should be given in aqueous solution, by the mouth.

Polishing Crowns.—To prevent marring a gold crown when polishing, wet the inside of the crown with soap solution, fill it with modeling composition, and, while the latter is still soft, thrust the end of a stick or instrument-handle into it. When the crown is finished, soften the composition in warm water and remove.—*Dental Hints.*

Cause for Toxic Action of Cocain Injections.—Dr. Maurel of Toulouse, France, claims that toxic effects are often caused by injecting the solution directly into the veins, causing the leucocytes to change form to a spherical form which prevents their passing freely through the capillaries of the lungs, where they form emboli which causes death. —*Cosmos.*

To Polish German Silver, Regulating Bands, Etc.—Pickle in hydrochloric acid and insert in a good sized piece of modeling compound which has been softened in the flame of a bunsen burner.

Cool and trim away excess of investment to expose the surface to be polished and use brick of tripoli with suitable brush wheels on lathe and finish with fine chalk or rouge.—*Dental Era*.

FUSIBLE ALLOY.—

| | | |
|---|---|---|
| ℞ Bismuth | - - - lbs. | 2 |
| Lead | - - - - " | 1 |
| Tin | - - - - " | 1 |
| Cadmium | - - - ozs. | 2 |

This alloy melts below the boiling point of water, and is tougher and shrinks less than most alloys now on the market.—H. Igel, in *Items of Interest*.

MENTHO-PHENOL.—Melt together one part of phenol crystals and three parts of menthol crystals, the result will be a pungent, aromatic amber-colored antiseptic mixture, which is soluble in alcohol, ether, chloroform and the essential oils, and is a good solvent of iodoform and aristol. A three per-cent aqueous solution makes a good mouth wash for infectious or ulcerative affections of the mouth. —Wm. Schaeffer, in *Medical Journal*.

NEW ROOT FILLING MATERIAL.—

| | |
|---|---|
| ℞ Whiting - - - | ℥ ss |
| Aristol - - - | grs. xij |
| Linseed Oil - - | q. s. |

Add to make a doughy consistence. Grind the Whiting and Aristol thoroughly together, sieve and then add the Oil until a thick mass is obtained, pack into root canal with probes or brooches covered with cotton, using small pellets at a time, cover with indestructible filling.—J. Levin, in *Dental Cosmos*.

TRAINING CHILDREN IN THE CARE OF THEIR TEETH. —The Louisville Dental College furnishes the principals of all the schools with printed slips, setting forth the fact that the teeth need certain care, and inspection by a dentist at frequent intervals and offering to perform such services at the college clinics free of all cost to such children as the principal may send with a statement that they are unable to have the work done by a regular practitioner.—Dr. W. E. Grant, in *Digest*.

Limitation in Cavity Preparation.—In the extension of cavity margins for prevention there are certain limitations; such as sensitive dentin, nervousness of patient and perhaps lack of time; but I do not believe we are ever justified in doing anything less than what we know to be right, simply because the patient can not afford to pay the fee to which we think we are entitled. Malpractice can scarcely be justified because of the patient's inability to render adequate compensation.—H. B. Tileston, in *Digest*.

To Facilitate taking Plaster Impressions for Partial Dentures.—Use a flat bottom tray and before putting the plaster in place fit in two pieces of metal plate bent to right angles so that the horizontal part lies on the bottom of the tray and the perpendicular part will stand upright and extending mesio-distally. These upright parts will remain in the plaster impression and will serve as line of cleavage in breaking the impression away from the teeth after the tray is removed.—E. L. Townsend, in *Pacific Gazette*.

A New Form of Vacuum Chamber.—When packing the vulcanite, carefully place a strip of semi-soft velum rubber one-eighth inch wide, around the edge of the metal chamber, and over both lay a piece of thin tin foil, extending to outer edge of the flexible rubber. Then finish packing as usual. After vulcanizing remove metal chamber and tin foil, this will give a flexible border to the air chamber, with a free edge or lip which keeps the contact when the plate is moved in mastication. It also relieves irritation in tender mouths.—Dr. J. A. Johnson, in *Items of Interest.*

To Facilitate Placing of Rubber Dam on Badly Decayed Teeth.—Prepare cavity with retaining grooves to cervical margin, fill the cavity largely with cotton well packed to place, leaving space for restoration of proximal contours with a quick setting amalgam, solidly packed to place over the cotton dressing. After the amalgam has hardened sufficiently, cut through to and remove the cotton. This will give direct and free access to the pulp chamber and permit easy medication, and will restore the tooth so that the rubber dam may be easily applied. This sort of treatment is also useful to prepare a tooth for an application of nerve paste or cocain by the pressure method.—O. E. Inglis, in *Itomatologist.*

Precaution in Using Hydrogen Dioxid.—Attention has been called to the ready absorbability of this substance and the consequent danger of fatal gaseous embolism caused by the bubbles of oxygen formed in the blood vessels after absorption. The oxyhemoglobin in the blood will absorb a small amount of oxygen without harm. Inflamed structures are particularly active in decomposing hydrogen dioxid and absorption is always slow from such tissues.

A neutral preparation is desirable when used where liable to be absorbed, and this never in stronger solutions than from eight to ten volumes. It may be neutralized by adding solution of sodium borate, drop at a time, until a neutral action results.—*Pacific Medical Journal.*

Collection Possible.—In the December *Digest*, page 1054, we stated that a dentist in Iowa recently lost a suit which he brought to enforce payment for services, because it was discovered that he was not licensed and had therefore violated the law when he did the work. A similar case came up last month in Wisconsin, where a dentist was suing a patient to recover payment for services rendered. The defendant's attorney discovered that the dentist did not receive his license until three months after the work was done, and so he was practising dentistry unlawfully. The court held that the statute merely imposed a penalty for practising dentistry without a license, but did not prohibit recovery for services rendered, and he ruled further that the testimony was not permissible under the pleadings.—*Digest.*

Stable Solutions of Cocain.—It is a well-known fact that chloretone is an antiseptic of much value. It has been found to be the ideal preservative of solutions of certain organic substances, notably cocain and its salts. As the result of a series of careful experiments we have brought out two solutions of cocain hydrochlorate with chloretone; one of two percent and the other of four percent strength.

These solutions not only remain clear for an indefinite time, but they retain their activity apparently unimpaired for months. They are always ready for use either locally or hypodermatically, and will supply a real need in minor surgery and dentistry.

Solution cocain hydrochlorate, two percent, with chloretone, is supplied in ounce vials at forty-two cents an ounce; the four percent solution is also supplied in ounce vials, at seventy cents an ounce.—*Therapeutic Notes*.

PERONIN AS A DENTAL ANESTHETIC.—This substance is known on the market by the name of *chlondrild*. It is a white, odorless powder, only slightly soluble in water. It accelerates respiration. In small doses it produces a sudden decrease of blood-pressure; in larger doses it causes death from arrest of circulation, the heart remaining in diastole.

Professor Bugolini has studied the anesthetic properties of peronin, and Dr. Juata has tried it in ophthalmic surgery. I have introduced it in dental surgery, and have found that it produces a profound and durable anesthesia. I use a one-half of one percent solution, in cold water, and almost without any exception the extractions are performed painlessly. The fluid should be injected deeply into the tissues, using all the care required by operations of this kind. Sometimes I use two percent solution, in hot water, hypodermically or by applying it to the gums for two or three minutes.

I have never observed any complications, nervous or otherwise, and, as its anesthetic effect lasts a long time, the patient never feels any post-operative pain.—Dr. Benvenuti, of Florence, Italy, in *Cosmos*.

Hypodermic Injection of Adrenalin to Control Hemorrhage in an Operation.—The region of the operation was anesthetized by Schleich's method, and one drachm of a 1-to-1,000 solution of Adrenalin Chlorid, in normal saline solution, was injected deep into the tissues, in two places half an inch apart. Ten minutes later an incision, one inch long and one inch deep, was made into the tissues. There was scarcely any hemorrhage, in fact only a little oozing of blood, not enough to obscure the parts. This operation was performed on a patient who had bled more than the average, in a location where, normally, there is considerable vascularity, while in this case congestion was present. The wound was packed with sterile gauze, and upon removing this the next day it was found unstained, except that portion which rested against the cut surface, showing that there had been no secondary hemorrhage.

It is interesting to note that as a result of the use of adrenalin the patient's pulse became very strong, but otherwise there was no systemic disturbance. The author has observed the beneficial effect of adrenalin upon the heart in cases where the drug has simply been sprayed into the nose, and has found it very effectual in counteracting the toxic effect of cocain.—F. C. Todd, M.D., in *Therapeutic Notes.*

The Painless and Antiseptic Treatment of Surgical and Traumatic Wounds.—An antiseptic dusting powder which is also locally anesthetic in its effect should be of great service to the hospital, railway, military and general surgeon. Borochloretone, which we have recently placed in the hands of the medical profession, is both antiseptic and anesthetic when applied to wounds of all kinds. It is superior to any other similar preparation in several particulars. It has an agreeable camphoraceous odor which is not so pronounced as to be objectionable. It does not become pasty, or form a hard crust over the surface of the wound; in other words, it retains its capacity for absorption. As it is an active germicide its use reduces the risk of septic infection to a minimum, and stimulates healthy and rapid healing. Finally, boro-chloretone is a local anesthetic—that is, it allays pain when applied to a wound. This feature especially adapts it to the treatment of severe painful injuries of any kind. After cleansing and closing the wound boro-chloretone is freely dusted over it in quantity sufficient to exclude the germ-laden air and take up the secretions. The dressing is changed daily, if necessary, to insure thorough cleanliness. The result in all cases reported is cessation of pain and soreness, and thorough and rapid healing.—*Therapeutic Notes.*

